# Quality Management for Point-Of-Care Testing of Pathogen Nucleic Acids: Chinese Expert Consensus

**DOI:** 10.3389/fcimb.2021.755508

**Published:** 2021-10-13

**Authors:** Xi Mo, Xueliang Wang, Zhaoqin Zhu, Yuetian Yu, Dong Chang, Xinxin Zhang, Dong Li, Fenyong Sun, Lin Zhou, Jin Xu, Hong Zhang, Chunfang Gao, Ming Guan, Yanqun Xiao, Wenjuan Wu

**Affiliations:** ^1^ Pediatric Translational Medicine Institute, Shanghai Children’s Medical Center, School of Medicine, Shanghai Jiao Tong University, Shanghai, China; ^2^ Department of Molecular Biology, Shanghai Centre for Clinical Laboratory, Shanghai, China; ^3^ Department of Laboratory Medicine, Shanghai Public Health Clinical Center, Fudan University, Shanghai, China; ^4^ Department of Critical Care Medicine, Ren Ji Hospital, School of Medicine, Shanghai Jiao Tong University, Shanghai, China; ^5^ Department of Laboratory Medicine, Shanghai Pudong Hospital, Fudan University Affiliated Pudong Medical Center, Shanghai, China; ^6^ Department of Infectious Diseases, Research Laboratory of Clinical Virology, Rui Jin Hospital, School of Medicine, Shanghai Jiao Tong University, Shanghai, China; ^7^ Department of Laboratory Medicine, Shanghai Tongji Hospital, School of Medicine, Tongji University, Shanghai, China; ^8^ Department of Clinical Laboratory Medicine, Shanghai Tenth People’s Hospital of Tongji University, Shanghai, China; ^9^ Department of Laboratory Medicine, Changzheng Hospital, Naval Medical University, Shanghai, China; ^10^ Department of Clinical Laboratory, Children’s Hospital of Fudan University, Shanghai, China; ^11^ Department of Clinical Laboratory, Shanghai Children’s Hospital, Shanghai, China; ^12^ Clinical Laboratory Medicine Center, Yueyang Hospital of Integrated Traditional Chinese and Western Medicine, Shanghai University of Traditional Chinese Medicine Shanghai, Shanghai, China; ^13^ Department of Laboratory Medicine, Huashan Hospital, Shanghai Medical College, Fudan University, Shanghai, China; ^14^ Department of Laboratory Medicine, Shanghai East Hospital, Tongji University School of Medicine, Shanghai, China

**Keywords:** pathogen, nucleic acid, point-of-care testing, expert consensus, whole-process operation management

## Abstract

COVID-19 continues to circulate globally in 2021, while under the precise policy implementation of China’s public health system, the epidemic was quickly controlled, and society and the economy have recovered. During the pandemic response, nucleic acid detection of SARS-CoV-2 has played an indispensable role in the first line of defence. In the cases of emergency operations or patients presenting at fever clinics, nucleic acid detection is required to be performed and reported quickly. Therefore, nucleic acid point-of-care testing (POCT) technology for SARS-CoV-2 identification has emerged, and has been widely carried out at all levels of medical institutions. SARS-CoV-2 POCT has served as a complementary test to conventional polymerase chain reaction (PCR) batch tests, thus forming an experimental diagnosis platform that not only guarantees medical safety but also improves quality services. However, in view of the complexity of molecular diagnosis and the biosafety requirements involved, pathogen nucleic acid POCT is different from traditional blood-based physical and chemical index detection. No guidelines currently exist for POCT quality management, and there have been inconsistencies documented in practical operation. Therefore, Shanghai Society of Molecular Diagnostics, Shanghai Society of Laboratory Medicine, Clinical Microbiology Division of Shanghai Society of Microbiology and Shanghai Center for Clinical Laboratory have cooperated with experts in laboratory medicine to generate the present expert consensus. Based on the current spectrum of major infectious diseases in China, the whole-process operation management of pathogen POCT, including its application scenarios, biosafety management, personnel qualification, performance verification, quality control, and result reporting, are described here. This expert consensus will aid in promoting the rational application and robust development of this technology in public health defence and hospital infection management.

## Introduction

The development of molecular biology technologies has enabled specific and sensitive detection of pathogen nucleic acids in a sample. Molecular testing has become one of the most important approaches for diagnosing infectious diseases, especially for detecting slow-growing pathogens that are difficult to cultivate under standard laboratory conditions as well as rare or newly emerging pathogens. For example, infection by SARS-CoV-2 is mainly assessed by nucleic acid testing. However, conventional molecular detection methods, such as regular PCR and qPCR testing, require specialized equipment and long batch test cycles and need to be carried out by professionals in a qualified gene amplification laboratory. Overall, aetiological diagnosis of acute or severe infection is often delayed in hospital outpatient and emergency centres and intensive care units (ICUs), thus delaying treatment and increasing the risk of nosocomial infection. In addition, a large number of patients visit fever clinics or emergency centres for observation during epidemics, resulting in serious shortages of consultation space, medical staff, and personal protective equipment. Accordingly, there is currently an urgent need to effectively shorten waiting and diagnosis time to improve medical care for infectious diseases and clinical microbiology.

Point-of-care testing (POCT), also known as “bedside testing” and “proximity testing”, refers to testing performed near or at the patient’s location, the results of which may lead to changes in treatment (ISO 22870, 2016). POCT has been widely used in medical and health institutions at all levels, mainly for the detection of blood biochemical and immunological indicators such as blood glucose and myocardial markers. In the field of infectious disease screening, POCT provides more rapid and sensitive diagnoses than traditional pathogenic detection methods. In general, POCT provided to patients in primary health care institutions and those receiving public health emergency treatment plays an extremely important role in the prevention and control of infectious diseases and hospital infection management. POCT can also help to improve health economic benefits as well as public health management. Moreover, the combination of molecular biology technology and POCT not only integrates sample “extraction-amplification-detection” but also has advantages of portability, easy operation, rapidity, airtightness, and suitability for a variety of scenarios while ensuring high specificity and sensitivity (Kuupiel et al., 2017; Larkin and Garner, 2020).

As a new detection technology with great potential, POCT nucleic acid detection eliminates many steps for specimen processing and large-scale equipment detection and simplifies the tedious process of data processing, and thus can directly and quickly provide reliable results for guiding patient treatment. Therefore, whether it is used in emergency responses to major public health incidents or in the diagnosis, treatment, and management of nosocomial infections, POCT can provide important technical support and guarantee precision treatment as well as scientific prevention and control.

## Scenarios for the Application of POCT

In hospital outpatient and emergency centres and paediatric laboratories, POCT has been used for a long time to detect the antigens of or antibodies against pathogens such as influenza, diarrhoea-causing pathogens, and group A *Streptococcus*. Although such examinations are simple to perform and the rapid results have enabled timely treatment, the detection sensitivity and specificity of these methods are lower than those of molecular detection techniques (Basile et al., 2018; Nelson et al., 2020; Song et al., 2021; Wang et al., 2021). Therefore, examinations based on antigen or antibody detection are prone to missing or misdiagnosing pathogens. In addition, during acute respiratory infection epidemics, false negatives in antigen testing occur due to the high prevalence of the pathogen in the population; as such, pathogen nucleic acid testing should be performed (Harris et al., 2018; Patel and Suh-Lailam, 2019; Nichols et al., 2020).

Pathogen nucleic acid POCT is mainly employed in fever clinics, emergency centres, paediatrics departments, and laboratories in certain wards. As opposed to routine physical and chemical index POCT detection, pathogen nucleic acid POCT first requires assessment of the hazard degree of the biological factor, after which examinations can be carried out under conditions that meet the biosafety requirements. The main applications of different pathogen nucleic acid POCT detection technologies differ according to the current technical conditions and future technology development trends. Recommendations for scenarios of POCT applications are shown in **Table 1**.

**Table 1:** Application scenarios of pathogen nucleic acid POCT.

Laboratory	Purpose	Examples of pathogens detected
Emergency	Initial diagnosis and treatment	Group A *Streptococcus*, influenza
Fever clinics^*^	Diagnosis, treatment, and monitoring	Malaria, dengue fever, group A *Streptococcus*, SARS-CoV-2 and other respiratory viruses, atypical pathogens
Paediatric outpatient and emergency	Diagnosis, treatment, and monitoring	Malaria, dengue fever, group A *Streptococcus*, and pathogens causing respiratory tract infections, rash syndrome, and diarrhoea syndrome
Laboratory/third-party laboratory	Diagnosis, treatment, and monitoring	^*^TB, HIV, HBV, HCV, dengue fever, malaria, group A *Streptococcus*, central nervous system infection pathogens, bloodstream infection pathogens, respiratory tract infection pathogens, multi-drug resistant bacteria
Specific wards^*^	Treatment and monitoring	Central nervous system infection pathogens, bloodstream infection pathogens, respiratory tract infection pathogens, multi-drug resistant bacteria

^*^1. Fever clinics: Pathogen nucleic acid POCT projects carried out in these facilities mainly target fever and respiratory infection pathogens such as SARS-CoV-2, influenza A virus, influenza B virus, and respiratory syncytial virus.

2. Specific wards refer to wards in a hospital with specific prevention and control requirements, such as infectious disease wards and ICUs under closed-loop management of designated hospitals.

3. TB, Mycobacterium tuberculosis; HIV, human immunodeficiency virus; HBV, hepatitis B virus; HCV, hepatitis C virus.

As POCT results are medical test reports, the reports must be accurate and timely, with complete information. Due to the nature of POCT, it can be carried out in the laboratory of a non-clinical testing centre (medical laboratory) by a well-trained person without clinical examination certificates. However, considering the high infectivity and pathogenicity of some pathogens, the requirement for the appropriate laboratory biosafety level and the technologists’ qualifications need to follow the regulations of the countries or regions located, and fully consider the actual conditions, including the pathogen risk grade, the waiting time and intensiveness of outpatient and emergency patients, *etc.* Training for POCT-performing technologists includes instrument operation, specimen collection, reagent selection, quality assurance, instrument calibration, maintenance and troubleshooting, biosafety management, and medical waste treatment. Only after the training is completed and exams are passed can technologists carry out POCT examinations and issue reports. Except medical laboratories, whether technologists in one department can perform tests and issue reports for another department should follow the rules of hospital’s management committee. The POCT management committee of the hospital authorizes the corresponding work of technologists and conducts management and supervision.

## Biosafety Management

Pathogen nucleic acid POCT should be carried out in compliance with national and local biosafety regulations and hospital infection management regulations (Order No. 380 of the State Council of the People's Republic of China, 2011; Health Industry Standard of the People's Republic of China, 2017; Order No. 424 of the State Council of the People's Republic of China, 2018; The State Council of the People's Republic of China, 2020). The molecular POCT system is an integrated closed system, which means that the nucleic acid extraction, amplification, and detection steps all occur in an enclosed space, which effectively prevents biological samples and genetic materials from being released into the environment. Therefore, nucleic acid POCT poses a relatively small risk to personnel performing the tests. Nevertheless, because of direct contact with patients and samples during sample collection and sample addition to the reagent card or pouch, there is still a risk of pathogen exposure. Therefore, for highly infectious pathogens, such as SARS-CoV-2, the equipment necessary for biosafety level 2 laboratories or above are recommended, including biosafety cabinets and autoclaves, to ensure that pre-treatment of specimens with infection risk (such as liquefaction of sputum specimens and sub-packaging of specimens) is performed safely. To reduce the risk of aerosol formation, spillage, or exposure, patient specimens should be handled in the manner recommended by the product manual.

Fever clinics should meet the regional requirements with three areas (the clean area, the semi-contamination area, and the contamination area) and two aisles (the medical staff aisle and the patient aisle). The clean area mainly include medical and nursing rest areas, which should have independent entrances and exits. The semi-contamination area (also called buffer zone) mainly includes an area for unloading contaminated protective equipment and storage warehouses for disinfection materials. The contamination area mainly includes medical functional areas such as consultation rooms, wards, laboratories, and disposal rooms. The medical functional area should make full use of information technology, such as patient self-service machines for registration, appointment, payment, and printing testing reports, *etc.*, to reduce the waiting time and the risk of cross-infection during the diagnosis and treatment process. In general, pathogen nucleic acid POCT should be carried out in a laboratory in a medical functional area.

Furthermore, the testing area should be kept clean and orderly to prevent cross-contamination. The surface and floor should be disinfected every day as well as immediately after spillage or visible contamination. Technologists should wear personal protective equipment correctly in accordance with the requirements of the biosafety laboratory, including disposable gloves, which should be replaced between runs. In addition, test reagents must be stored and handled according to product instructions.

Supervision and verification of POCT safety management measures should be consistent with the current requirements for infectious pathogen gene amplification laboratories, including following guidelines for the preservation, use, and destruction of samples containing suspected infectious pathogens; laboratory biosafety operations; and disinfection and sterilization of laboratory exhaust gas, wastewater, and waste disposal.

## Specimen Collection and Pre-Treatment

Specimen collection and pre-treatment for pathogen nucleic acid POCT involve the patient as well as sample collection and testing personnel. Improper handling during one or more of the steps may lead to disqualification of samples before analysis and ultimately lead to incorrect test results. Recommendations for the common types of clinical specimens and their application are shown in **Table 2**.

**Table 2:** Common types of clinical specimens and their application.

Type of infection	Specimen type	Examples of pathogens	Main purpose
Multi-drug-resistant bacteria	Sputum, stool, anal swab, nasal swab	Carbapenem resistance gene (CRE), Methicillin-resistant *Staphylococcus aureus* (MRSA)	Multi-drug-resistant bacteria control
Gastrointestinal tract infection	Stool, anal swab	*Clostridium difficile*	Detection of *Clostridium difficile* infection
Respiratory tract infection	Throat swab	Group A *Streptococcus*	Detection and identification of the aetiology of pharyngitis
	Nasopharyngeal swab	Acute respiratory viruses (influenza A virus, influenza B virus respiratory syncytial virus, SARS-CoV-2)	Used for the diagnosis of acute respiratory infections, guiding treatment, and assisting infection control
	Sputum/alveolar lavage fluid/tissue	*Mycobacterium tuberculosis* and rifampicin resistance Viruses, fungi, atypical pathogens	Screening and diagnosis of lower respiratory tract infections, guiding medication
Genitourinary tract infection	Urine, pus, cervical specimen, swab	*Chlamydia trachomatis*, Mycoplasma, *Neisseria gonorrhoeae*, HPV, GBS*	Screening and diagnosis of sexually transmitted diseases
Blood-borne infection	Blood	HIV, HBV, HCV*	Surgery/blood transfusion/haemodialysis/endoscopy preparation
Central nervous system infection	Cerebrospinal fluid	EV71, Japanese encephalitis virus, cryptococci, *Mycobacterium tuberculosis*	Diagnosis of central nervous system infection

*GBS, group B Streptococcus; HBV, hepatitis B virus; HCV, hepatitis C virus; HIV, human immunodeficiency virus; HPV, human papilloma virus.

### Selection of the Sample Collection Tube and Preservation Buffer

If one-step nucleic acid extraction is used in pathogen nucleic acid POCT, specific preservation buffer components in the sample collection tube may affect the extraction and amplification efficiency, resulting in a decrease in detection sensitivity. Thus, the sample collection tube and preservation buffer provided by the manufacturer should be used for specimen collection. Additionally, the performance of the sample collection and preservation matching the detection system should be verified before clinical use.

Should the laboratory decide to use a collection tube and buffer not recommended by the manufacturer, it is incumbent on the lab to determine that the performance of the test is not adversely altered by using that sample collection tube and buffer prior to implementation. Under special circumstances, if a sample collection tube and preservation buffer not provided with the manufacturer’s testing reagents are needed, the performance of the collection tube and buffer should be verified before sample collection.

### Collection and Pre-Treatment of Specimens

The main types of specimens collected for pathogen nucleic acid POCT are throat swabs, nasal swabs, nasopharyngeal swabs, blood, cervical specimens, sputum, urine, stool, cerebrospinal fluid, puncture fluid, and tissues. The collection methods of various specimens should be consistent with the requirements for conventional molecular biology testing.

Specimen collection method, selection of specimen type, specimen stability, transport temperature and time, acceptance standards, and storage conditions should strictly follow product instructions.

After the specimens are collected, examinations should follow operating instructions. Labelling of the specimens should be kept consistently during the specimen processing procedure when more than one tubes or containers need to be used. Each of these tubes or containers must be labelled with the patient identifying information, including the final testing device, to ensure that the final result is reported for the appropriate patient. Liquefaction of sputum, faeces, tissues, and other specimens must be performed according to manufacturer’s instructions. Moreover, special attention should be given to the amount of specimens used for testing, which should specifically follow the manufacturer’s instructions. A lesser amount of specimen may give a false negative result due to inadequate target nucleic acids being present. The use of a larger amount of specimen recommended by the manufacturer can also result in a false negative result due to the introduction of impurities and inhibitors of nucleic acid amplification.

## Performance Verification of Testing Programmes

Before routine application, the laboratory should conduct independent performance verification of the POCT system to verify that its performance is consistent with declared performance indicators (International Organization for Standardization, 2012a; International Organization for Standardization. 2015; International Organization for Standardization, 2016; International Organization for Standardization, 2020; Verbakel et al., 2020). If any situation that seriously affects the analytical performance of the testing programme occurs, for example, when the main components (such as the fluorescence channel) have been repaired or replaced, performance should be verified before the testing programme is reactivated (International Organization for Standardization, 2012a; International Organization for Standardization, 2016).

Pathogen nucleic acid POCT mainly includes qualitative and quantitative pathogen detection, and different performance parameters are focused for different examinations (International Organization for Standardization, 2010; International Organization for Standardization, 2015; Clinical and Laboratory Standards Institute, 2018). The performance indicators that need to be verified for qualitative testing include, at a minimum, the accuracy, precision (repeatability and reproducibility), and limit of detection (International Organization for Standardization, 2008). The performance indicators that need to be verified for quantitative testing include, at a minimum, the trueness, precision, linearity interval, and limit of quantification (International Organization for Standardization, 2014a). For nucleic acid detection of multiplex pathogens, it is advisable to test for as many common pathogen types and genotypes as possible, especially testing different genotypes and strains of the target pathogens detected by the assay (International Organization for Standardization, 2018).

### Performance Verification of Qualitative Detection

#### Accuracy

To calculate the accuracy, at least five negative samples and generally no less than 10 positive samples (which should include weak positive samples), should be selected. If a sufficient number of positive samples cannot be obtained, a simulated sample can be prepared manually; if a weak positive sample is difficult to obtain, a similar sample can be obtained by appropriately diluting a positive sample. Negative samples should contain nucleic acid sequences that share homology with the nucleic acid sequence of the test subject and are likely to cause the same or similar clinical symptoms. After performing the examination according to the procedure, the results should be compared with known test results to calculate the coincidence rate, and an accuracy ≥ 90% indicates passing verification (International Organization for Standardization, 2008).

#### Precision (Repeatability and Reproducibility)

To calculate system precision, negative and weak positive samples (fresh or cryopreserved) should be prepared. If a sufficient number of clinically positive samples cannot be obtained, positive samples can be diluted appropriately, or simulated samples can be manually prepared. In accordance with the testing procedure, the test should be performed three times a day for three consecutive days (if multiple individuals perform the test, a different person should perform it each time). For multi-channel POCT equipment, each test channel should be tested at least once. Test results for negative and weakly positive samples should be exactly the same (International Organization for Standardization, 2008; International Organization for Standardization, 2014b).

#### Limit of Detection (LoD)

To calculate LoD, a fixed-value reference substance (such as an international reference, national reference, and manufacturer reference) should be diluted to the LoD concentration declared by the manufacturer; the measurement should be repeated 5 times or 20 times in different batches (*e.g.*, measurement performed over 5 days, with 4 samples measured per day). For five repeated tests, 100% of the target nucleic acid must be detected; for 20 tests, the target nucleic acid must be detected at least 19 times (International Organization for Standardization, 2012b; Wang et al., 2020).

### Performance Verification of Quantitative Detection

#### Trueness

One method for measuring trueness is to select at least two concentrations of a reference material (such as certified reference materials, trueness control products, and external quality assessment samples for trueness verification) according to the measurement interval of the POCT system required for verification. The measurement should be repeated three times for the standard substance samples at each concentration, with the mean value compared with the calibrated concentration value to assess bias.

Another method for measuring trueness is to collect at least 20 clinical samples from patients with a clear clinical diagnosis; the pathogen titres in these samples should cover the linear detection range of the POCT system as much as possible. The collected samples can be tested on the same day or within a week. After all experiments are completed, the test results are compared with the original test results, and bias is determined. At least 80% of sample results should have bias less than ± 7.5% (International Organization for Standardization, 2014a).

#### Precision

Fresh or frozen clinical samples can be used to measure precision. When the analyte in the sample is unstable or the sample is difficult to obtain, a sample with a matrix similar to the actual sample (such as a quality control) may be used. Imprecision (coefficient of variation) of at least two samples should be evaluated, and the concentration of the target nucleic acid in the selected sample should be within the measurement interval of the POCT system required for verification. When appropriate, the concentration in at least one sample should be approximately the medically determined level. Each sample should be tested 3–5 times a day for 5 consecutive days. After all experiments are finished, statistical analysis is performed to calculate intra-run and inter-run precision, which are compared with those in the manufacturer’s instructions to judge whether the results are acceptable (International Organization for Standardization, 2014a; International Organization for Standardization, 2014b).

#### Linear Range (When the Manufacturer’s Manual Provides the Performance Index)

To calculate the linear range, a clinical sample in which the concentration of the target nucleic acid is close to the upper limit of the linear range should be selected and diluted to 5~7 concentrations with a normal human negative sample at a tenfold ratio, covering the limit of quantification (lower limit and upper limit). All samples are tested in the same batch, and each concentration is tested at least three times. The mean value of the test results of each sample is calculated separately, and outliers are eliminated. Using the calculated dilution value as the theoretical value, regression analysis is performed on the actual measured and theoretical values for each diluted sample. Performance meeting the linearity requirement stated in the manufacturer’s manual is considered acceptable (International Organization for Standardization, 2020b).

#### Limit of Quantification

To determine the limit of quantification, a standard substance with a known value is diluted to the lower LoD stated in the instructions, and the measurement is repeated at least 20 times. After the experiment is completed, the results of each sample are compared with the reference value of the sample. Bias between the test results (converted to log values) and the reference value should be less than ± 7.5%; if n = 20. It is necessary that ≥ 19 test results meet the above requirements (International Organization for Standardization, 2012b; Wang et al., 2020).

## Quality Assurance Measures

The laboratory should establish and implement standard operating procedures for the entire testing process, including but not limited to specimen collection, transport, storage, specimen reception and pre-treatment, testing operations and re-testing procedures, result reporting and interpretation, instrument and equipment maintenance, performance verification, internal quality control (IQC) and external quality assessment (EQA) (International Organization for Standardization, 2011; International Organization for Standardization, 2012a; International Organization for Standardization, 2012c; International Organization for Standardization, 2016). Qualified laboratories can establish an individualized quality control plan (IQCP) (Centers for Disease Control and Prevention (U.S.), 2015). When the laboratory uses multiple or multiple brands of POCT instruments for routine operation, it is necessary to follow the hospital’s POCT management documents and conduct regular inter-instrument comparisons (International Organization for Standardization, 2016).

### Internal Quality Control (IQC)

The laboratory should carry out IQC to monitor the stability of the testing process. IQC procedures should be developed, and specific measures for preventing contamination during nucleic acid testing should be included (International Organization for Standardization, 2012a; International Organization for Standardization, 2016).

In POCT, an internal control (internal standard) is usually included for each sample, and an external quality control (should include positive and negative samples) is run as a separate sample. Detection of the internal control indicates that nucleic acids were extracted correctly from patient samples, which is a necessary step to obtain correct results. External quality control products evaluate whether the testing system provides correct results. A complete IQC programme can control the POCT process, evaluate the performance of the testing system, and provide quality assurance.

IQC products should be equipped with negative control products (for monitoring contamination) and weak positive quality control products (to prevent false negatives). When the POCT system is used for the first time, the negative and weak positive quality control products should be tested first, and clinical sample examination should be started after quality control is complete. Thereafter, the negative and weak positive controls should be routinely evaluated according the local rules or actual situation. For example, in China, the negative and weak positive controls need to be evaluated every 24 hours or 50 samples (depending on the number of specimens processed). For multiple nucleic acid detection systems, it is recommended that as many types of common pathogens as possible be included in evaluation. One or more pathogens can be included in one round of reagent detection, but should rotate all of the target pathogens on repeated quality control testing runs.

Quality control data for qualitative testing items need to match expected negative and positive results; it is recommended that the weak positive quality control results for quantitative testing items be judged using the Westgard multi-rule approach. A report can be issued only when quality control is passed. Moreover, procedures should be developed to analyse the failure of quality control, and corresponding measures should be taken. The impact of quality control failure on the results of previous patient samples should also be checked.

### External Quality Assessment (EQA)

Laboratories should participate in EQA activities to monitor the accuracy of the testing process (International Organization for Standardization, 2013; Sciacovelli et al., 2018; Kabugo et al., 2021). The personnel performing nucleic acid POCT examinations should use the same testing system to assess samples for EQA, and there should be regulations prohibiting comparing EQA results with other laboratories. Additionally, the laboratory should analyse unqualified EQA results, take corrective measures, and record them properly. If no EQA program is available for a specific analyte, laboratories should share clinical samples with another laboratory for testing. The laboratories will then compare the results from both facilities to ensure that they are the same. Such testing should be performed at least twice a year (International Organization for Standardization, 2012a).

## Analysis and Reporting of Results

The laboratory should analyse and interpret the results of tests according to manufacturer’s instructions. Although POCT equipment provides clear positive, negative, or invalid results, the laboratory still needs to refer to the background data (such as melting peak graphs and amplification curve graphs) when such data are available for comprehensive judgement regarding results.

When the POCT system is running well, the background data for test results (including internal references and pathogens to be tested) are correct and IQC is passed, the result is considered valid and the positive or negative results provided by the equipment can be reported.

Some POCT reagents will produce positive results for highly pathogenic microorganisms, such as *Vibrio cholerae*, SARS-CoV-2, *Mycobacterium tuberculosis*, and *Bordetella pertussis*. The laboratory should report the results to the sample-sending doctor as soon as possible after verifying the background data, and report to public health administration such as CDC for infectious disease if required. Regarding the management and control of major infectious diseases, reporting should be conducted in accordance with national laws, regulations, and management practices. In addition, some POCT reagents will produce positive results for drug resistance genes (such as MRSA, CRE, and rifampicin resistance) or highly virulent strains (such as the highly virulent *C. difficile* strain 027). In these cases, the laboratory needs to indicate the results of drug-resistance genes on the report after confirmation with the corresponding pathogen testing results. The samples should be re-examined if necessary, and the results should be compared with molecular test and microbial culture drug susceptibility results from the central laboratory (Mo et al., 2020; Wen et al., 2021).

The report of POCT results must be clearly marked with the words “POCT” and detection methods; the name of the POCT equipment and reagents used should also be indicated. When the report is released, the testing results should be entered into the laboratory information system (LIS), saved as a backup for inquiries, and connected to the hospital information system (HIS). In addition, the following aspects need to be considered when issuing examination reports:

- Since pathogen types are limited by POCT panels, the report should clearly indicate all pathogens tested and indicate whether a certain pathogen is detected or not detected. The report cannot simply state that the sample is “virus not detected” or “bacterium not detected”.
- As nucleic acid testing is usually unable to distinguish between viable and non-viable pathogens or between colonization and active infection, a positive result only indicates that the DNA or RNA of a certain pathogen is currently present in the sample and does not mean that disease symptoms are caused by the pathogen. Because POCT is usually based on specific primers that target a certain pathogenic microorganism, there may be false negatives due to the lack of amplification of the sequence when the sequence of the pathogen in this region is mutated, which does not indicate that the pathogen is not present. Therefore, it is recommended to state the above information on the report sheet, and clinicians also need to make comprehensive judgements based on the actual clinical situation and other examination results.

Re-examination is required when the following situations occur: i) quality control fails, and the result is invalid; ii) background data do not match the POCT result (*e.g.*, the background data have obvious amplification peaks, but POCT reports a negative result because of the threshold or grey area problems); iii) POCT results are inconsistent with routine laboratory examination results (especially for positive results for highly infectious pathogens), and the clinician believes that re-examination is necessary. If the same results are obtained from re-examination, the laboratory should fully communicate with the clinician the existing test results and reasons and then decide whether to collect a new sample for examination.

## Limitations of POCT Nucleic Acid Detection

- The detection sensitivity of pathogen nucleic acid POCT is higher than that of immunological POCT (Basile et al., 2018; Nelson et al., 2020; Song et al., 2021; Wang et al., 2021), and the use of inexperienced personnel in molecular detection carries the risk of detection failure and environmental cross-contamination (Wiencek and Nichols, 2016). For example, in a clinic or public space where the flu vaccine or SARS-CoV-2 vaccine is administered, contaminated equipment will produce false positives. In view of the simplicity of molecular POCT operation, by following the manufacturer’s instructions, the chance of contamination and human error will be minimized.
- Although the POCT instrument provides clear positive, negative, or invalid results, the platform is usually not connected to LIS; hence, the results must be entered manually, and there is a risk of human error in data entry.
- At present, reagents for pathogen nucleic acid POCT are more expensive than those for conventional fluorescent quantitative PCR detection and antigen/antibody-based detection. Although nucleic acid POCT has higher sensitivity and specificity than immunological POCT (Basile et al., 2018; Nelson et al., 2020; Song et al., 2021; Wang et al., 2021), it is still necessary to consider rapid antigen-antibody screening in economically underdeveloped areas where molecular diagnosis is not affordable.
- Although molecular POCT instruments are usually small and portable, detection throughput is usually low. Indeed, some instruments can only run one to two samples at a time. In fever clinics and emergency departments of large hospitals or emergency care clinics, multiple instruments are often needed to effectively meet the requirements for pathogen detection flux.

In general, pathogenic nucleic acid POCT has gradually entered routine operation in the diagnosis, treatment, and management of infectious diseases and has unique advantages. Whether in a general hospital laboratory or in a fever clinic, POCT can greatly reduce the pressures and challenges brought by centralized sample delivery. Therefore, standardizing POCT management is an urgent task that benefits both doctors and patients, and it is necessary to incorporate POCT into the entire experimental detection system. The reliability of results will be increased through systematic quality control and quality assurance measures. Overall, pathogen nucleic acid POCT provides strong support for improving national medical quality and safety and the public health emergency response, standardizing the rational use of antimicrobial drugs, and establishing a hierarchical diagnosis and treatment model for infectious diseases.

## Author Contributions

WW, YX, MG, and CG initiated the consensus. XM, XW, ZZ, YY, and WW drafted the consensus. All authors contributed to the article and approved the submitted version.

## Funding

This work was supported by the Shanghai Public Health System Construction Three-Year Action Plan (2020 to 2022) Key Disciplines grant (GWV-10.1-XK04), Shanghai Key Laboratory of Clinical Molecular Diagnostics for Pediatrics (20dz2260900), Shanghai Excellent Technology Leader (20XD1434500), Scientific and Technology Commission of Shanghai Municipality (20Y11903600), and Shanghai Municipal Health Commission (2019SY049).

## Conflict of Interest

The authors declare that the research was conducted in the absence of any commercial or financial relationships that could be construed as a potential conflict of interest.

## Publisher’s Note

All claims expressed in this article are solely those of the authors and do not necessarily represent those of their affiliated organizations, or those of the publisher, the editors and the reviewers. Any product that may be evaluated in this article, or claim that may be made by its manufacturer, is not guaranteed or endorsed by the publisher.

## Abbreviations

CRE, carbapenem resistance gene; EQA, external quality assessment; GBS, group B *Streptococcus*; HBV, hepatitis B virus; HCV, hepatitis C virus; HIS, hospital information system; HIV, human immunodeficiency virus; HPV, human papilloma virus; ICU, intensive care unit; IQC, internal quality control; IQCP, individualized quality control plan; LIS, laboratory information system; MRSA, methicillin-resistant *Staphylococcus aureus*; PCR, polymerase chain reaction; POCT, point-of-care-testing; TB, *Mycobacterium tuberculosis*.
